# Adaptations to Submarine Hydrothermal Environments Exemplified by the Genome of *Nautilia profundicola*

**DOI:** 10.1371/journal.pgen.1000362

**Published:** 2009-02-06

**Authors:** Barbara J. Campbell, Julie L. Smith, Thomas E. Hanson, Martin G. Klotz, Lisa Y. Stein, Charles K. Lee, Dongying Wu, Jeffrey M. Robinson, Hoda M. Khouri, Jonathan A. Eisen, S. Craig Cary

**Affiliations:** 1 University of Delaware, Lewes, Delaware, United States of America; 2 University of Delaware, Newark, Delaware, United States of America; 3 University of Louisville, Louisville, Kentucky, United States of America; 4 University of California Riverside, Riverside, California, United States of America; 5 University of Waikato, Hamilton, New Zealand; 6 University of California Davis, Davis, California, United States of America; 7 The J. Craig Venter Institute, Rockville, Maryland, United States of America; Institute for Genome Sciences, University of Maryland School of Medicine, United States of America

## Abstract

Submarine hydrothermal vents are model systems for the Archaean Earth environment, and some sites maintain conditions that may have favored the formation and evolution of cellular life. Vents are typified by rapid fluctuations in temperature and redox potential that impose a strong selective pressure on resident microbial communities. *Nautilia profundicola* strain Am-H is a moderately thermophilic, deeply-branching *Epsilonproteobacterium* found free-living at hydrothermal vents and is a member of the microbial mass on the dorsal surface of vent polychaete, *Alvinella pompejana*. Analysis of the 1.7-Mbp genome of *N. profundicola* uncovered adaptations to the vent environment—some unique and some shared with other *Epsilonproteobacterial* genomes. The major findings included: (1) a diverse suite of hydrogenases coupled to a relatively simple electron transport chain, (2) numerous stress response systems, (3) a novel predicted nitrate assimilation pathway with hydroxylamine as a key intermediate, and (4) a gene (*rgy*) encoding the hallmark protein for hyperthermophilic growth, reverse gyrase. Additional experiments indicated that expression of *rgy* in strain Am-H was induced over 100-fold with a 20°C increase above the optimal growth temperature of this bacterium and that closely related *rgy* genes are present and expressed in bacterial communities residing in geographically distinct thermophilic environments. *N. profundicola*, therefore, is a model *Epsilonproteobacterium* that contains all the genes necessary for life in the extreme conditions widely believed to reflect those in the Archaean biosphere—anaerobic, sulfur, H_2_- and CO_2_-rich, with fluctuating redox potentials and temperatures. In addition, reverse gyrase appears to be an important and common adaptation for mesophiles and moderate thermophiles that inhabit ecological niches characterized by rapid and frequent temperature fluctuations and, as such, can no longer be considered a unique feature of hyperthermophiles.

## Author Summary

Extreme environments, such as deep-sea hydrothermal vents, found 2,500 meters below the ocean surface, support large macrofaunal communities via microbially mediated carbon fixation processes using chemicals (chemoautotrophy) rather than light (photoautotrophy). The genome of one such model chemoautotrophic microbe, *N. profundicola*, was sequenced and described in this work. *N. profundicola*, distantly related to the pathogenic *Helicobacter* and *Campylobacter* species, contains a number of genes and pathways predicted to be important in DNA repair, environmental sensing, and metabolism, which are novel to either its subdivision or to all microbes. The genes and deduced metabolic pathways include several hydrogen uptake and release systems as well as a novel predicted nitrogen assimilation pathway. One gene involved in DNA repair, *reverse gyrase*, was thought to be a hallmark protein in hyperthermophiles, which are microbes that grow above 80°C. We found this gene to be highly expressed at 65°C, over 20°C above the optimal growth temperature of this organism. Therefore, the genome of this model deep-sea hydrothermal vent chemoautotroph may reflect what is required for life in an extreme environment, hypothesized to be similar to early earth conditions.

## Introduction

Food webs at deep-sea hydrothermal vents are based on microbial primary productivity fueled by chemical reactions rather than light. Microorganisms that thrive in these environments must adapt to fluctuations in temperature and redox conditions, ranging from the hot, sulfidic, heavy metal-laden plume at the vent outlet to cold, oxic seawater in the surrounding region [1]–[4]. In addition, DNA damaging agents such as ionizing radiation add to the harsh conditions with which hydrothermal residents must contend [5]–[8]. While a variety of diverse organisms have been isolated from hydrothermal environments [9],[10], it is clear from molecular surveys and *in situ* hybridization studies that *Epsilonproteobacteria* are numerically dominant and therefore likely key players in the cycling of C, N, and S at deep-sea hydrothermal vents [11].

Vent-associated *Epsilonproteobacteria* are diverse, including the deeply-branching *Nautiliales* and *Nitratiruptor* groups, *Sulfurospirillum*, *Arcobacter*, and the “*Thiovulgaceae*” Marine Groups I and II [11],[12]. Molecular and culture-based surveys have demonstrated *Epsilonproteobacteria* in diverse sub-seafloor habitats [13]–[17]. Two complete genome sequences of cultured vent *Epsilonproteobacteria* are available, *Nitratiruptor sp.* SB155-2 and *Sulfurovum* sp. NBC37-1 [18], while that of *Caminibacter mediatlanticus*, isolated from the Mid-Atlantic Ridge [19] has been sequenced to draft level. We describe here the 1.7 Mbp genome of *Nautilia profundicola* strain Am-H, the first from a member of the order *Nautiliales* isolated from hydrothermal vents [20],[21].

*N. profundicola* strain Am-H was originally isolated from the biomass of an *Alvinella pompejana* episymbiont community collected at 13°N along the East Pacific Rise Axial Caldera [20],[21]. Strain Am-H is only found in some episymbiont communities and is readily detected as a free-living organism in various mats on vent chimneys [20]. *N. profundicola* falls within the *Nautiliales* order of the *Epsilonproteobacteria* class, the deepest branching order of this subdivision [20], [22]–[25] and is closely related to other vent isolates: *Caminibacter hydrogeniphilus* and *Nautilia lithotrophica* [22],[23]. All are moderately thermophilic strict anaerobes that grow lithoautotrophically with H_2_ and elemental sulfur (S^0^). Most likely, all members of this group utilize the reductive or reverse tricarboxylic acid (rTCA) cycle for CO_2_ fixation [19],[21],[26]. *N. profundicola* and relatives differ from vent isolates whose genomes have been sequenced, including *Thiomicrospira crunogena* (*Gammaproteobacteria*), *Nitratiruptor sp.* SB155-2 (*Epsilonproteobacteria*), and *Sulfurovum* sp. NBC37-1 (*Epsilonproteobacteria*) [18],[27]. These are sulfur-oxidizing microaerophiles that fix CO_2_ via the Calvin-Benson-Bassham cycle (*T. crunogena*) or the rTCA cycle (*Nitratiruptor sp.* SB155-2, *Sulfurovum* sp. NBC37-1) [18], [27]–[29]. Am-H and *N. lithotrophica* are differentiated from *C. hydrogeniphilus* as well as other *Epsilonproteobacteria* (*Caminibacter mediatlanticus*, *Wolinella succinogenes*, and *Sulfuromonas denitrificans*) by the inability to respire nitrate [18],[19],[30],[31]. However, strain Am-H and *N. lithotrophica* can both utilize nitrate as the sole source of nitrogen [21],[23].

The large temperature and redox variations present in diffuse hydrothermal flow regimes, which may be extant examples of an early life environment [4], impose strong selective pressures for characteristic adaptations in resident microbial communities. Determining these exact mechanisms is one of the most compelling issues for vent microbiologists and others interested in adaptations to life in extreme environments. Prior surveys found that typical DNA-repair genes are absent from the *Nitratiruptor sp.* SB155-2 and *Sulfurovum* sp. NBC37-1 genomes [18], perhaps indicating that *Epsilonproteobacteria* genomes can rapidly change, which may confer a selective advantage in either pathogenic associations or deep-sea hydrothermal vent environments [18],[32],[33]. However, this flexibility must be balanced against DNA damage–inducing conditions present at deep-sea hydrothermal vents, including high, fluctuating temperatures and ionizing radiation.

A comparison of the *N. profundicola* genome to the genomes of other hydrothermal vent bacteria has suggested several common features of a hydrothermally adapted lifestyle. One example of such adaptation, a reverse gyrase, was further examined in terms of regulated expression in *N. profundicola*, distribution among other *Epsilonproteobacteria*, and expression in deep- sea hydrothermal environments. In addition, its genome sequence indicates that strain Am-H utilizes nitrate in the absence of a canonical nitrite reductase and instead likely relies on a novel pathway where hydroxylamine is a key intermediate. Our analyses suggest that not only is *N. profundicola* the deepest branching epsilonproteobacterial genome to be sequenced to date, but its genome may provide evolutionary insights beyond what is required for growth at hydrothermal vents.

## Results/Discussion

### General Genome Properties

**Figure 1:**
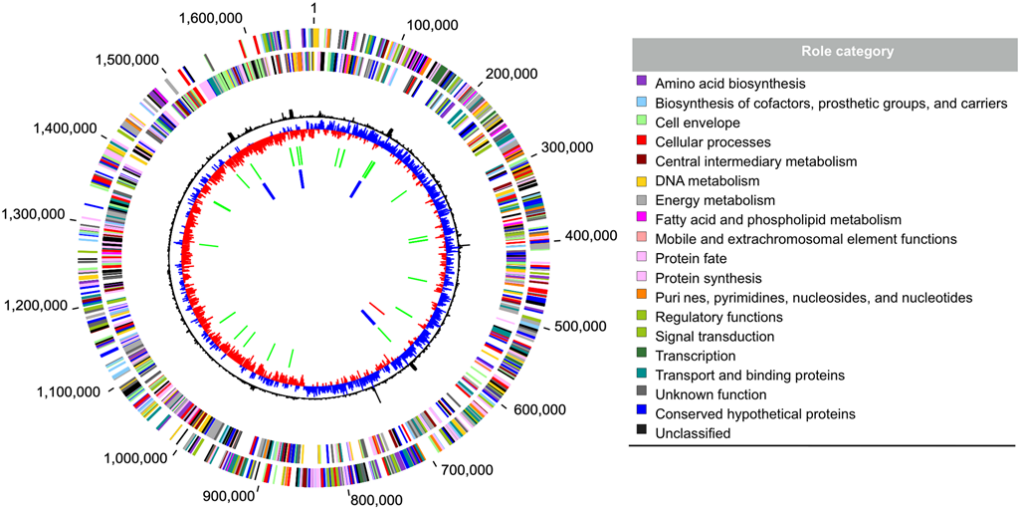
Circular View of the *N. profundicola* genome. Circles correspond to the following features, starting with the outermost circle: (1) forward strand genes (2) reverse strand genes (3) χ^2^ deviation of local nucleotide composition from the genome average (4) GC skew (5) tRNAs (green lines) (6) rRNAs (blue lines) (7) small RNAs (red lines). Genes are colored according to their role categories.

The genome of *N. profundicola* (GenBank accession number, CP001279) is a single circular chromosome of 1,676,444 bp containing 1,745 protein-coding genes (CDSs); a small genome compared to other free-living *Epsilonproteobacteria* [18],[31]. The compactness of the genome is reflected in the absence of duplicated gene clusters. The genome's general features are summarized in Figure 1 and Table 1. Other aspects of the isolate and methods are detailed in Table S1. Basepair 1 was assigned upstream of *dnaA*, the chromosomal replication initiator protein (GAmH_0001). The origin of replication is likely to be located in this area based on the shift in GC skew pattern [34]. The sequences of genes in the four rRNA operons are 100% identical even through the intergenic regions. The genome contains the most tRNA genes (48) of all the sequenced *Epsilonproteobacteria* genomes, including one corresponding to selenocysteine (Table 1). To date, 21.5% of bacterial genomes contains at least one clearly identified selenocysteine-dependent enzyme [35].

**Table 1:** General features of *N. profundicola* and other sequenced autotrophic environmental *Epsilonproteobacteria*.

Feature	*Nautilia profundicola*	*Sulfurovum* sp. NBC37-1	*Nitratiruptor* sp. SB155-2	*Sulfuromonas denitrificans* DSM 1251 ATCC 33889	*Caminibacter mediatlanticus* TB-2 (Draft)
Environment	Vent	Vent sediment	Vent	Coastal Marine sediment	Vent
Electron donor	H_2_, formate	H_2_, S^2−^, S^0^, S_2_O_3_^2−^	H_2_, S^2−^, S^0^, S_2_O_3_^2^	HS^−^, S_2_O_3_^2^	H_2_
Electron acceptor	S^0^	O_2_, NO_3_^−^	O_2_, NO_3_^−^	NO_2_^−^, NO_3_^−^, O_2_	NO_3_^−^, S^0^
Carbon source	CO_2_, formate	CO_2_	CO_2_	CO_2_, formate	CO_2_
Growth temperature range (optimum) (°C)	30–55 (40–45)	10–37 (33)	37–65 (55)	N.D. (22)	45–70 (55)
Motility	+	−	+	−	+
Genome					
Complete genome size (bp)	1,676,444	2,562,277	1,877,931	2,201,561	1,663,618 (unfinished)
G+C percent	33.5%	43.8%	39.7%	34.5%	27%
Total number of CDSs	1,745	2,466	1,857	2,104	1,889
Percent coding	93.7%	90.1%	95.1%	92.7%93.8%	93%
Number of rRNA operons (16S-23S-5S)	4	3	3	4	ND
Number of tRNA genes	48	44	45	44	ND
CDSs with functional assignment	1,204 (69.1%)	49.4%	61%	1,566 (74.4%)	ND
Presence of gene:					
Reverse gyrase (COG1110)	+	−	+	−	+
DNA repair photolyase (COG1533)	+	−	+	−	+
DNA polymerase IV (family X, COG1796)	+	+	+	−	+

Data for other species were collected from the Comprehensive Microbial Resource (http://cmr.jcvi.org/tigr-scripts/CMR/CmrHomePage.cgi), the NCBI Entrez Genome (http://www.ncbi.nlm.nih.gov/sites/entrez?dbgenome), and previous publications [18],[19],[29],[31]. ND = not determined.

There is little evidence of recent widespread horizontal gene transfer (HGT) events into the genome of *N. profundicola*, based on % G+C ratio or tetranucleotide frequency anomalies (Figure 1). While no extrachromosomal elements were found, the genome contains several features that may indicate the existence of mobile genetic elements. The chromosome contains a group of genes annotated as a plasmid stabilization system gene (GAmH_0503) and a toxin/antitoxin gene pair (GAmH_0504/502) most closely related to the *relBE*-3 family of toxin/antitoxins [36],[37]. This family, when found on plasmids, prevents plasmid loss during replication. Integrated into the genome, they are predicted as stress response factors to starvation or other adverse conditions by inducing cell cycle arrest or programmed cell death [36]. The lack of homologous genes in other *Epsilonproteobacteria* suggests a recent HGT event to *N. profundicola*. No typical transposons were identified in the genome, although there is one potential transposase (GAmH_0909) with similarity to a gene in *C. mediatlanticus*, which also lacks other identifiable transposases. This contrasts with *Nitratiruptor sp.* SB155-2 and *Sulfurovum* sp. NBC37-1 genomes, which contain 5 and 14 predicted transposases, respectively [18]. There are no apparent full length prophages, although there are four genes identified as phage integrases, three of which are putative site-specific recombinases (GAmH_0249, 1143, 0597, XerD), and one (GAmH_1472) with no significant similarities to genes in GenBank (e-value >0.005). One potential prophage, around 10 Kb in length, was identified using Prophage Finder [38]. This putative prophage spans 15 coding sequences (GAmH_1141-1155), including one of the phage integrases (GAmH_1143) and nine hypothetical proteins. Also in this region are a zonular occludens toxin (Zot) family protein (GAmH_1150), a bacteriophage replication gene A protein (GPA) (GAmH_1152), a putative cupin domain protein (GAmH_1153), a putative tetratrico peptide repeat (GAmH_1154), and DNA primase (GAmH_1155). The similarity of genes in this region to those found in other *Epsilonproteobacteria* indicates either an ancient phage integration event into an ancestral genome or that similar viruses infect the subdivision. Two tRNAs are coded within this potential prophage region, one of which is the only glutamine tRNA in the genome. The apparent lack of foreign DNA may be due to yet undescribed defensive mechanisms in *N. profundicola* or to the harsh external environment, which could select against the persistence of free DNA.

### Signal Transduction

Free-living bacteria must sense and respond to environmental changes quickly and do so through a variety of signal transduction mechanisms. Pathogenic species have fewer signal transduction proteins than their free-living counterparts, even when genome size is taken into account [39], as is the case among sequenced *Epsilonproteobacteria*. *N. profundicola*, *Nitratiruptor* sp. SB155-2, and *Sulfurovum* sp. NBC37-1 each have over 100 signal transduction genes, while most strains of *Campylobacter* and *Helicobacter* have about half as many, most of which code for histidine kinases, response regulators, or methyl-accepting chemotaxis proteins. Free-living *Epsilonproteobacteria* have many genes containing diguanylate cyclase (GGDEF) and phosphodiesterase (EAL) domains, whereas *Campylobacter* and *Helicobacter* genomes have only a few or none.

Among vent *Epsilonproteobacteria*, *N. profundicola* has the most stand-alone GGDEF and EAL domain family protein gene homologs, while *Nitratiruptor sp.* SB155-2 and *Sulfurovum* sp. NBC37-1 have more gene homologs belonging to a predicted signal transduction protein family containing a membrane domain as well as GGDEF and EAL domains (Table 2). *N. profundicola* is the only vent *Epsilonproteobacterium* that also has the HD-GYP domain, another phosphodiesterase. Given its smaller genome size and comparatively limited metabolism, its numerous coding sequences for cyclic diguanylate signaling relative to other vent organisms seems counterintuitive. Perhaps having more of these intracellular signaling molecules allows *N. profundicola* to more precisely or rapidly regulate protein expression in the highly variable vent environment. Then again, this may also reflect *N. profundicola*'s changing lifestyles between a free-living existence and being associated with a complex metazoan-microbial symbiosis system which is essentially a biofilm, and the different requirements for gene expression under these two conditions.

**Table 2:** Abundance of common signal transduction domains in the genomes of hydrothermal vent *Epsilonproteobacteria*.

*Domain*	*Nautilia profundicola*	*Caminibacter mediatlanticus*	*Nitratiruptor sp. SB155-2*	*Sulfurovum sp. NBC37-1*
histidine kinases*	12	11	13	29
response regulators*	15	17	19	30
GGDEF (COG2199)	18	9	9	5
EAL (COG2200)	13	7	10	4
HD-GYP (COG2206)	2	0	0	0
GGDEF+EAL (COG5001)	3	2	12	9

*: Signal transduction histidine kinases include COGs 0642, 0643, 3920, 4191, 4564 and response regulators include COGs 0745, 2197, 2204, 3706.

The sheer abundance of GGDEF and EAL domains within many genomes, including *N. profundicola*, suggests that a wide variety of environmental stimuli may be detected by these domains [40]. Both the two-component and c-diGMP signal transduction systems sense and respond to periplasmic substrates, and *N. profundicola* has GGDEF or EAL domains associated regions that bind amino acids (PBPb domain) or carbohydrates (CelB domain). In addition, as a strict anaerobe, *N. profundicola* requires the ability to detect oxygen and move away from it, and unsurprisingly possesses a PAS domain (common domain in **P**eriod circadian protein, **A**ryl hydrocarbon receptor nuclear translocator, and **S**ingle-minded protein) protein (COG 2202) that senses redox conditions and binds gaseous molecules like NO, CO, and O_2_ [41]. Known outputs of the c-diGMP system include changes in motility, virulence, heavy metal resistance, phage resistance, cell to cell communication, exopolysaccharide production, and biofilm formation [40],[42]. In the hydrothermal vent environment, *N. profundicola* would benefit from the regulation of motility and heavy metal resistance through c-diGMP molecules. In addition, regulation of phage resistance could be particularly important to *Epsilonproteobacteria* which lack traditional DNA repair systems.

### Cell Sensing Systems

*Nautilia profundicola* has homologs of the autoinducer-2 (AI-2) system of quorum sensing, S-ribosylhomocysteinase (*luxS*) (GAmH_0429), and 5′methylthioadenosine/S-adenosylhomocysteine nucleosidase (*pfs*) (GAmH_0316). At the time of writing, 22 out of 26 sequenced *Epsilonproteobacteria* have the *luxS* and *pfs* genes, with only four *Campylobacter* species lacking the AI-2 system. While LuxS has been shown to affect biofilm formation, motility, type III secretion, and the production of virulence factors and toxins in pathogens, it remains unclear whether AI-2 is truly a universal signal or simply a metabolic byproduct for most bacterial strains [43]–[45]. In support of the widespread nature of LuxS/Pfs in *Epsilonproteobacteria*, the metagenome of the *A. pompejana* episymbionts contains 36 *luxS* sequences and 49 *pfs* sequences [46]. The abundance of these genes in *Epsilonproteobacteria* at hydrothermal vents may offer a mechanism through which this group dominates episymbiont communities. Alternatively and perhaps more likely, quorum sensing at hydrothermal vents could be used for diffusion sensing [47]. The ability to detect diffusion rates in highly variable flow regimes could allow *N. profundicola* to regulate secretion of extracellular enzymes. Since the AI-2 inducer is a metabolic byproduct, loss of AI-2 to the environment is less energetically costly than the loss of an enzyme.

### Biofilm-Related Pathways: Polyamine, Flagellar, and Pilin Syntheses

*N. profundicola* likely maintains a mostly attached lifestyle due to the dynamic fluid flow and mixing occurring at vents. It is associated with some, but not all, episymbiont communities of the hydrothermal vent polychaete, *A. pompejana*, and is also associated with bacterial mats on chimney surfaces [20]. Polyamines have been shown to play significant roles in the formation of biofilms in *Yersinia pestis*, the causative agent of bubonic plague [48]. *N. profundicola* appears to be similar to *Y. pestis* and *H. pylori* in polyamine synthesis pathways [48],[49]. Genes encoding arginine decarboxylase (*speA*, GAmH_0346) and spermidine synthase (*speE*, GAmH_0033) are found in the *N. profundicola* genome for spermidine formation, but the conversion from agmatine to putrescine is likely performed jointly by agmatine deiminase (*aguA*, GAmH_1708) and *N*-carbamoylputrescine amidohydrolase (*aguB*, GAmH_0211) instead of agmatinase (*speB*, GAmH_0211). Spermidine may also be synthesized from carboxyspermidine by carboxynorspermidine decarboxylase (*nspC*, GAmH_0102), which hints at norspermidine production in *N. profundicola*. Norspermidine plays a significant role in *Vibrio cholerae* biofilm formation [50], and a homolog of *mbaA* (GAmH_1695), a norspermidine-regulated biofilm maintenance protein containing GGDEF and EAL domains in *V. cholerae* [51], is present in the *N. profundicola* genome. However, a homolog of *nspS*, which encodes a norspermidine-binding regulatory protein in *V. cholerae* that mediates biofilm formation, was not identified in the *N. profundicola* genome.

Findings from a recent survey of genes critical for biofilm formation in *E. coli* reinforced the theory that genes related to surface structures (i.e., flagella and pilin) play major roles in such processes [52]; similar findings have been reported for *C. jejuni* [53]–[55]. The highly motile *N. profundicola* has genes for 56 flagellar and pilin proteins, four chemotaxis proteins, and 11 methyl-accepting chemotaxis proteins. While *N. profundicola* has most of the same flagellar genes as found in *Nitratiruptor* sp. SB155-2, the distribution of genes varies between the two organisms. *Nitratiruptor* flagellar genes are all found within the same region of the genome which has a distinct G+C content [18], while *Nautilia* flagellar genes are distributed throughout the genome and are most closely related to flagellar genes in other *Epsilonproteobacteria*.

### Detoxification and Resistance Pathways

There are several genes in *N. profundicola* and other vent *Epsilonproteobacteria* that encode proteins directly or peripherally involved in resistance to toxins. One of these is a member of the Nudix family of proteins that clean the cell of toxic nucleotide metabolites [56]. This gene (GAmH_1363) is predicted to encode ADP-ribose pyrophosphatase and is found only in the vent epsiloproteobacterial and *Arcobacter butzleri* genomes and not in pathogenic *Epsilonproteobacteria* [18],[57]. The protein confers tellurite resistance in both *Rhodobacter sphaeroides* and *Methanococcus jannaschii*, presumably through detoxification of ADP-ribose, which non-enzymatically derivatizes terminal amino groups, lysines, and cysteines of proteins [58]–[60].

*N. profundicola* has two genes encoding proteins homologous to arsenate reductase genes, one of which (GAmH_1558) is homologous to members of the YffB subfamily of arsenate reductases that reduces arsenate to arsenite via reducing equivalents from glutathione. Similar genes are found in the other three vent *Epsilonproteobacteria* as well as *W. succinogenes* and *A. butzleri*. All the vent *Epsilonproteobacteria* as well as *S. denitrificans* and *A. butzleri* contain homologs to the second arsenate reductase (GAmH_0397 in *N. profundicola*). *W. succinogenes*, *Sulfurovum*, *Nitratiruptor*, and *S. denitrificans* contain an apparent operon that encodes an arsenite permease and regulatory protein, ArsR [18],[31],[61], whereas both *N. profundicola* and *C. mediatlanticus* lack an arsenical permease homolog (COG1055), but contain an *arsR* homolog (GAmH_0374) elsewhere. Therefore, these microbes may not detoxify arsenic or may use an as yet unrecognized mechanism for arsenite transport.

The genome contains two types of cytochromes predicted to be involved in protection against O_2_ and H_2_O_2_: cytochrome *bd* (*cydBA*, GAmH_0086, 87), is used to reduce O_2_ to H_2_O, and cytochrome *c*_551_ peroxidase (GAmH_0241 and 0648) detoxifies peroxide. The genome does not contain predicted genes for catalase or superoxide dismutase, which may be involved in defense against oxidative stress in *S. denitrificans* [31]. However, *N. profundicola* has a homolog of alkylhydroperoxide reductase (Ahp, GAmH_0523) that can scavenge endogenous hydrogen peroxide [62], as well as a predicted peroxide stress regulator (GAmH_0524) which is most closely related to sequences in other *Epsilonproteobacteria*. The genome also contains 2 homologs of methionine sulfoxide reductases (*msrA*, GAmH_1068 and *msrB*, GAmH_0900) predicted to repair oxidative damage.

All bacteria are thought to employ low molecular weight thiols as a component of oxidative stress resistance. These systems are typified by biosynthetic pathways for the thiol (i.e., glutathione or mycothiol), enzymes that utilize the reduced form of thiol as a reductant, and an enzymatic system for reduction of the oxidized disulfide form of the thiol. The *N. profundicola* genome does not encode a recognizable pathway for biosynthesis of typical redox balancing thiols or a cognate disulfide oxidoreductase. As noted above, *N. profundicola* contains a potential arsenate reductase that in other organisms requires glutathione as a reductant. Either the *N. profundicola* version of this enzyme utilizes a different reductant or *N. profundicola* must synthesize glutathione by a currently unrecognized pathway. Glutathione might be obtained from the environment, but this has only been reported for *Streptococcus mutans* [63]. Furthermore, we are unaware of any gene encoding a glutathione transporter in any microbial genome.

Coenzyme A (CoA) could serve as an alternative to redox balancing thiol since *N. profundicola* encodes a potential CoA-disulfide oxidoreductase (GAmH_0923). CoA has been suggested as an alternative to glutathione in the hyperthermophilic archaeon *Pyrococcus furiosus*, based on high levels of intracellular CoA [64] and the presence of a CoA-disulfide reductase (CoADR) activity (PF1186) [65]. Recently, however, PF1186 was found to possess NAD(P)H:polysulfide oxidoreductase activity, and the transcript was positively regulated by the addition of elemental sulfur to *P. furiosus* cultures [66]. This suggests that the CoADR activity could be a side reaction, with a primary function in sulfur metabolism as described below.

### Carbon Metabolism

**Figure 2:**
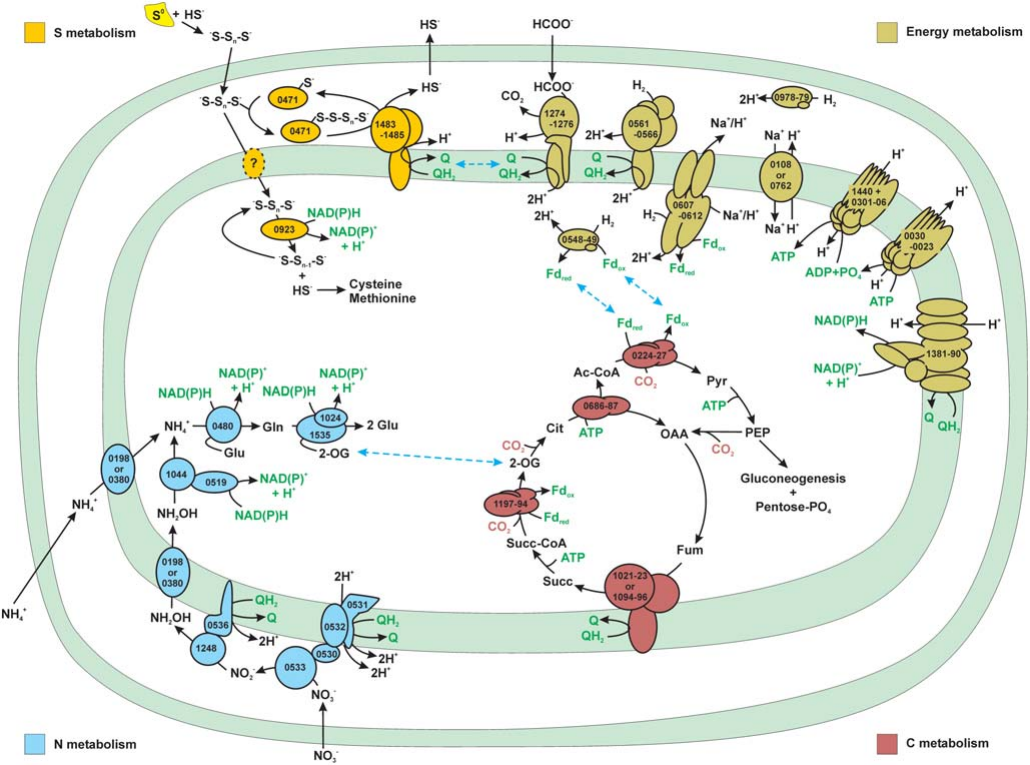
Major metabolic subsystems in *N. profundicola* AmH as deduced from the genomic sequence. Proteins that belong to a given subsystem are color coded as follows: energy metabolism, olive green; carbon (C) metabolism, pale red; nitrogen (N) metabolism, light blue; sulfur (S) metabolism, yellow. Numbers denote the ORFs encoding the enzymes predicted to catalyze a particular reaction. Individual subunits are not drawn to scale. Common energy and reductant carriers shared across subsystems are depicted in green type. Interactions between subsystems based on shared pools of metabolites or reductant carriers are indicated by the dashed blue double arrows. For details on each subsystem, please refer to the appropriate section of the text.

*N. profundicola* was the first chemolithoautotrophic *Epsilonproteobacteria* isolated from deep-sea hydrothermal vents [20] and was hypothesized early on to use the rTCA cycle for carbon fixation [26]. Enzymatic evidence, carbon isotopic fractionation patterns, and the genome sequence data presented in this paper all strongly support this hypothesis (Figure 2, cell model) [21]. Like *S. denitrificans*, *N. profundicola* encodes two putative fumarate reductase/succinate dehydrogenase (Fdr/Sdh) complexes. One of the complexes (GAmH_1023-1021) is similar to the vent epsilonproteobacterial and *S. denitrificans* Fdr/sdhABC complexes, the other (GAmH_1096-1094) is more similar to the second copy in *C. mediatlanticus* as well as those in the *Beggiatoa* sp. PS (whole-genome amplified and pyrosequenced partial sequence), Aquificales, and Chlorobium genomes [67],[68],[69]. Unlike *S. denitrificans*, both complexes in the *N. profundicola* genome contain a putative membrane-anchoring subunit. The genome contains homologs of all of the genes necessary for the generation of 5-carbon sugars via the pentose phosphate pathway as well as the genes necessary for both glycolysis and gluconeogenesis.

Of the sequenced vent *Epsilonproteobacteria*, only *N. profundicola* can grow with formate as a carbon source, but it cannot utilize the C4 dicarboxylic acids (i.e., fumarate or succinate) [18],[19],[21] (Table 1). Therefore, it was surprising to find three homologs to genes encoding C4-dicarboxylate transporter proteins (GAmH_0376, 0470 and 0768) as well as two putative operons with genes encoding a C4-TRAP system for ATP-independent transport of C4 compounds (GAmH_1457-1459, 0113-0115), also found in *C. mediatlanticus* [70]. The closest homolog to one of the C4-TRAP system operons is from *Psychromonas ingrahamii* 37, a marine *Gammaproteobacterium* isolated from polar sea ice [71]. Further experiments are required to determine the functionality of C4 usage in *N. profundicola*.

### Sulfur Metabolism

**Figure 3:**
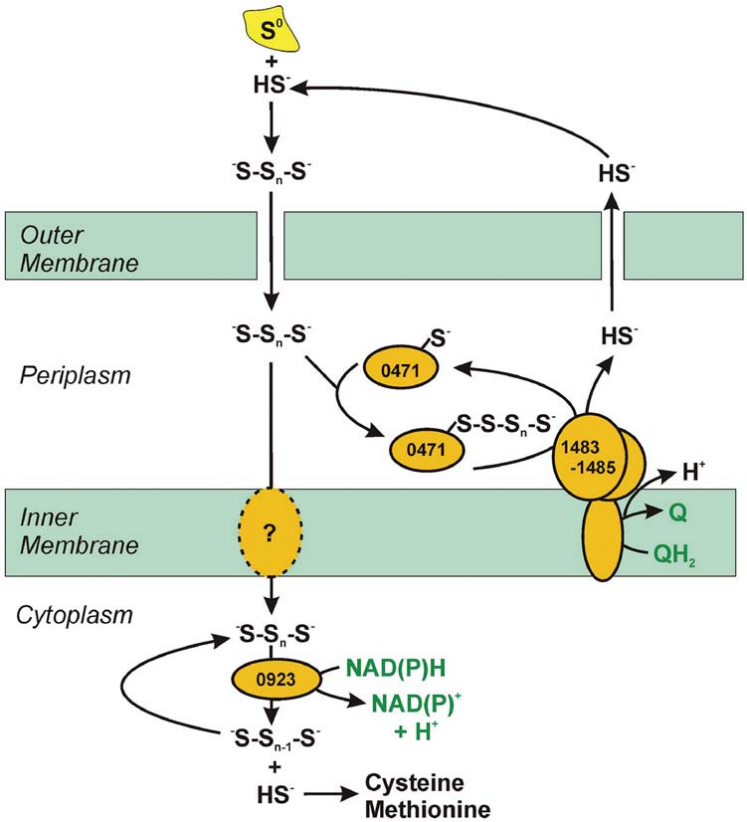
Deduced pathways for sulfur metabolism in *N. profundicola* AmH. Enzymes are labeled with the number of the ORF that encodes the protein. The central intermediate in the pathway is periplasmic polysulfide, which is predicted to have two fates. First, polysulfide can be used as the terminal electron acceptor for energy conservation by a quinol oxidizing polysulfide reductase (right branch, 1483–1485). This process likely involves a polysulfide carrier (0471) similar to the Sud protein of *Wolinella succinogenes*. Second, polysulfide may be assimilated after transport after conversion to sulfide by an NAD(P)H:polysulfide oxidoreductase (center branch, 0923). This is the only route for the synthesis of cysteine and methionine as AmH lacks a reductive sulfate assimilation pathway.

Sulfur serves two critical purposes for *N. profundicola*: as the terminal electron acceptor for energy conservation and as a source of sulfide for biosynthesis. *N. profundicola* utilizes only elemental sulfur/polysulfides, not sulfate or thiosulfate, as an electron acceptor and presumably must conserve energy in the form of proton motive force from this activity. Three enzymatic systems for the reduction of elemental sulfur have been described: membrane-bound polysulfide reductase [72], cytoplasmic sulfhydrogenase [73],[74], and a soluble cytoplasmic NAD(P)H:polysulfide oxidoreductase [75]. Membrane-bound polysulfide reductase is exemplified by the *Wolinella succinogenes* PsrABC enzyme that serves as the terminal oxidase for electron transport from H_2_ or formate to polysulfide via a quinone. The periplasmic enzyme Sud can accelerate this process by functioning as a polysulfide-binding protein that presents the substrate for reduction by the molybdopterin-containing PsrABC [76]. Phenotypically, *N. profundicola* should contain this enzyme system ,and homologs of the PsrABC system are indeed encoded by GAmH_1483-1485 (Figures 2 and 3). *N. profundicola* would presumably use the polysulfides formed from the reaction of S^0^ and sulfide, which readily occurs at hydrothermal vents and are also found in the culture medium [20]. A homolog of the Sud protein is encoded elsewhere (GAmH_0471). The *N. profundicola* genome also encodes multiple hydrogenase homologs and one presumptive formate dehydrogenase (GAmH_1274-1276) that forms the input end of an electron transport chain terminating at the PsrABC enzyme.

For biosynthesis, all organisms must either obtain sulfur-containing amino acids from the environment or synthesize them *de novo* by producing and capturing sulfide. In contrast to many bacteria, *N. profundicola* lacks a sulfate transporter and reductive sulfate assimilation pathway to produce sulfide in the cytoplasm. However, *N. profundicola* does contain genes that correspond to O-acetyl-serine sulfhydrylase (OAS, GAmH_1543) and O-acetyl-L-homoserine sulfhydrylase (OAHS, GAmH_0859) that capture sulfide for cysteine and methionine biosynthesis, respectively. Based on the genome sequence, we propose that *N. profundicola* utilizes NAD(P)H polysulfide oxidoreductase (GAmH_0923) to produce sulfide from polysulfide in the cytoplasm for assimilation. GAmH_0923 was noted above as a potential CoA-disulfide oxidoreductase involved in oxidative stress resistance. Clearly, GAmH_0923 is a high priority target for further biochemical and regulation studies to determine whether its primary role in *N. profundicola* is to serve as a terminal electron acceptor, a mediator of thiol redox balance, or both. A similar lack of reductive sulfate assimilation genes coupled with the presence of sulfide assimilation genes was found in the genome of *Thiomicrospira crunogena* [27], a vent-associated microaerobic sulfur-oxidizing chemoautotroph, suggesting that this may be a common trait of organisms inhabiting sulfide rich environments.

### Nitrogen Metabolism

**Figure 4:**
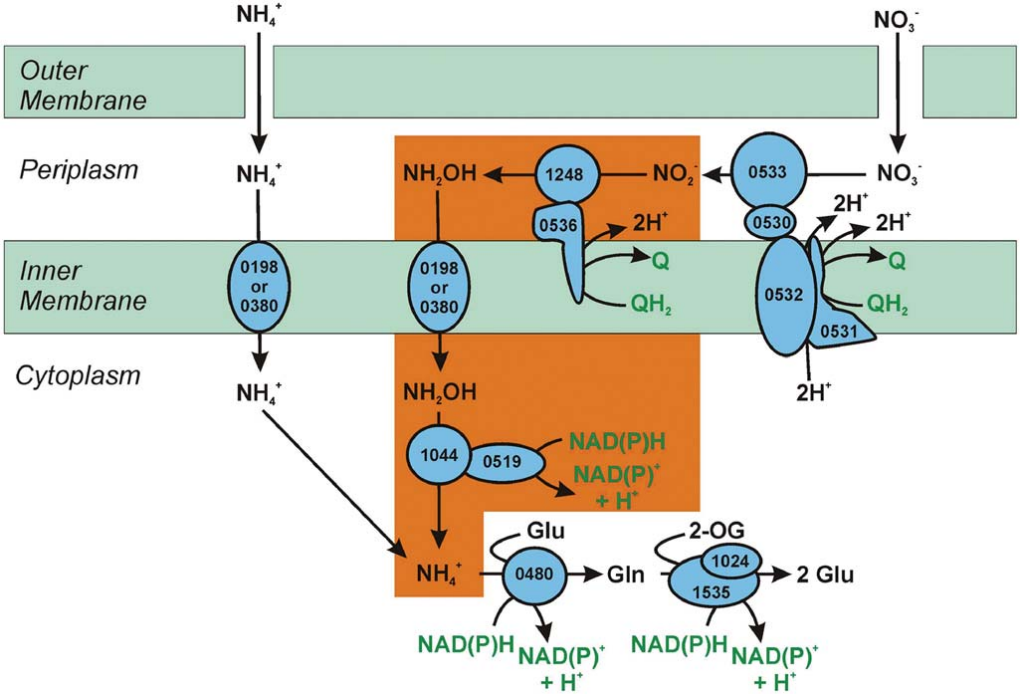
Deduced pathways for nitrogen metabolism in *N. profundicola* AmH. Enzymes are labeled with the number of the ORF that encodes the protein. The proposed alternative pathway for nitrite reduction to ammonia, predicted to be essential for the growth of AmH on nitrate, is highlighted in orange. The first step of this pathway consists of a hydroxylamine:ubiquinone redox module (HURM) composed of a novel nitrite reductase (1248; reverse hydroxylamine oxidoreductase) that produces hydroxylamine utilizing electrons donated from the (ubi)quinol pool via a cytochrome *c* protein (0536) in the NapC/NrfH/cM552 protein superfamily. Following transport of hydroxylamine into the cytoplasm, the second step is the reduction of hydroxylamine to ammonia by a hybrid cluster protein/hydroxylamine reductase (1044) utilizing reducing power from a flavin-containing NADH oxidase (0519).

*N. profundicola* grows chemolithotrophically on hydrogen with ammonium or nitrate, but not with urea, as a sole N source [21]. Further growth experiments demonstrated similar specific growth rates (0.059 h^−1^ vs. 0.044 h^−1^) on nitrate and ammonium, respectively. However, morphological observations showed elongation and filament production atypical of normal growth in the nitrate-grown cells (data not shown). Genes encoding a typical ammonia transport and assimilation pathway are present: two AmtB transporters (GAmH_0198 and GAmH_0380), NADPH-glutamate synthase (GAmH_1535/ GAmH_1024), and glutamine synthetase (GAmH_0480) (Figures 2 and 4) [77]. As *N. profundicola* cannot utilize nitrate as a terminal electron acceptor, the presence of genes encoding dissimilatory periplasmic nitrate reductase (*napAGHBFD*, GAmH_0533-0527) is quite surprising. The *nap* operon displays high sequence similarity to those encoding periplasmic nitrate reductase in *Wolinella succinogenes* [30] and *Sulfurimonas denitrificans* [31]. Periplasmic nitrate reductase functions in both organisms solely in nitrate respiration and appears to be coupled to nitrite processing systems: in *W. succinogenes* to the ammonia-forming respiratory pentaheme cytochrome *c* nitrite reductase, NrfAH [30], and in *S. denitrificans* to the NO-forming cytochrome *cd_1_* nitrite reductase, NirS [31]. However, *N. profundicola* lacks homologs of NrfA, NirS, or any other nitrite reductase, nor are homologs of nitrite transporters (NarK, FocA, etc.) present. Taken together, these results suggest that *N. profundicola* must utilize a novel pathway for nitrite processing as well as nitrogen assimilation. We propose that *N. profundicola* utilizes a heretofore unrecognized nitrogen assimilation pathway that relies on the concerted actions of the periplasmic nitrate reductase, a hydroxylamine ubiquinone redox module (HURM, [78]), and NADH-dependent hydroxylamine reductase (hybrid cluster protein, Har [79]). The *N. profundicola* HURM system consists of hydroxylamine oxidoreductase (GAmH_1248) and a cytochrome c_M_552-like protein (GAmH_0536), while Har is encoded by GAmH_1044.

In aerobic and anaerobic ammonia-oxidizing bacteria, HURM functions as a quinone reductase mediating the oxidation of hydroxylamine and hydrazine [80]. In contrast, we propose that the *N. profundicola* HURM functions in opposite direction and similar to NrfAH as a periplasmic quinol oxidase system that reduces nitrite to hydroxylamine (Figure 4). Recent studies revealed that octaheme cytochrome *c* hydroxylamine reductase has evolved from pentaheme cytochrome *c* nitrite reductase, NrfA [80], via the intermediate octaheme cytochrome c nitrite reductase, ONR [78],[81]. If structural features whose evolution have been identified as the crucial event for the conversion of the once N-oxide reductase into an N-oxide dehydrogenase (oxidase) are absent from a given Hao protein sequence, a reverse function of Hao as a nitrite reductase is feasible [82]. Among these structural features missing in the deduced protein sequence of *N. profundicola* Hao is the critical tyrosine residue (Figure S1), which serves as the protein-derived cross-link to catalytic heme P460 in functional hydroxylamine oxidoreductase functional enzyme complex (HAO) trimers, which is also missing in the NrfA and ONR proteins [81]. Recent work by Pacheco and associates [83] confirmed that even fully functional hydroxylamine oxidoreductase from *Nitrosomonas europaea* (a chemolithoautotrophic ammonia oxidizer) can catalyze the reduction of nitric oxide to ammonia given the presence of a proper redox partner. We propose that the *c*_M_552-type tetracytochrome *c* (GAmH_0536) could serve as such suitable redox partner to reverse HAO (GAmH_1248), which needs to be experimentally tested. The uncharged hydroxylamine produced by reverse HAO in the periplasm likely readily diffuses across the plasma membrane, either by simple diffusion or facilitated through ammonia or other major facilitator protein channels. It has been shown that hydroxylamine accumulates readily in the anammoxosome of *Kuenenia stuttgartiensis* [84], a structure to be reached by external hydroxylamine only after permeation across the plasma membrane. Uptake studies of hydroxylamine into this anaerobic ammonia-oxidizing planctomycete have also been performed [85]. Once in the cytoplasm, hydroxylamine as a powerful mutagen poses imminent danger and needs to be detoxified as it arrives. This is likely accomplished by NADH-dependent Har, which reduces hydroxylamine to ammonia using electrons served by a flavin-containing NADH oxidoreductase (GAmH_0519). Intriguingly, the promoter region of the *har* gene contains a conserved recognition motif (CATTGACcgcaGTCAATG) for the FNR-like transcriptional repressors, NnrR and DNR, that are responsive to nitrite and/or nitric oxide [86]. This suggests regulation of at least the *har* gene by nitrosative intermediates.

The *N. profundicola* Hao- and c_M_552-encoding genes are in the vicinity of genes that encode critical functions in the cytochrome *c* maturation system II of *N. profundicola*: GAmH_1250, *ccd*A; GAmH_0538, *res*B. Also, the *hao* gene is adjacent to gene encoding a small monoheme cytochrome *c* (GAmH_1249) homologous to cytochromes *c*552 (M.G. Klotz, unpublished results), which serve as redox carriers in the respiratory chains of many chemolithotrophs and are known to redox-partner with complexes III and IV [27],[31],[87]. The *N. profundicola* genome does not encode a complex III, and complex IV is a cytochrome D ubiquinol oxidase (GAmH_0086-89). Because the predicted catabolic modules in the periplasm interact directly with the quinone pool (Psr, FDH, uptake-hydrogenase), cytochrome c552 likely connects both or one of the encoded cytochrome *c*551 peroxidases (GAmH_0241, GAmH_0648) to c_M_552, thereby providing hydrogen peroxide tolerance and an additional respiratory electron sink to the quinone pool. A putative redox interaction between cytochromes c552 and c_M_552 has been discussed in the literature ([78] and references therein).

We have preliminary evidence to suggest that at least three genes in the pathway, *napA*, *hao*, and *har*, are upregulated in *N. profundicola* cells grown in the presence of nitrate vs. ammonium. These experiments are ongoing and will be published at a later date. This pathway may also be present in other bacteria, at least in other *Nautilia* strains. The genome sequence of *Nautilia lithotrophica* is currently being sequenced, and other *Nautilia* strains are also being tested for growth on nitrate as an N source.

### Energy Metabolism

**Figure 5:**
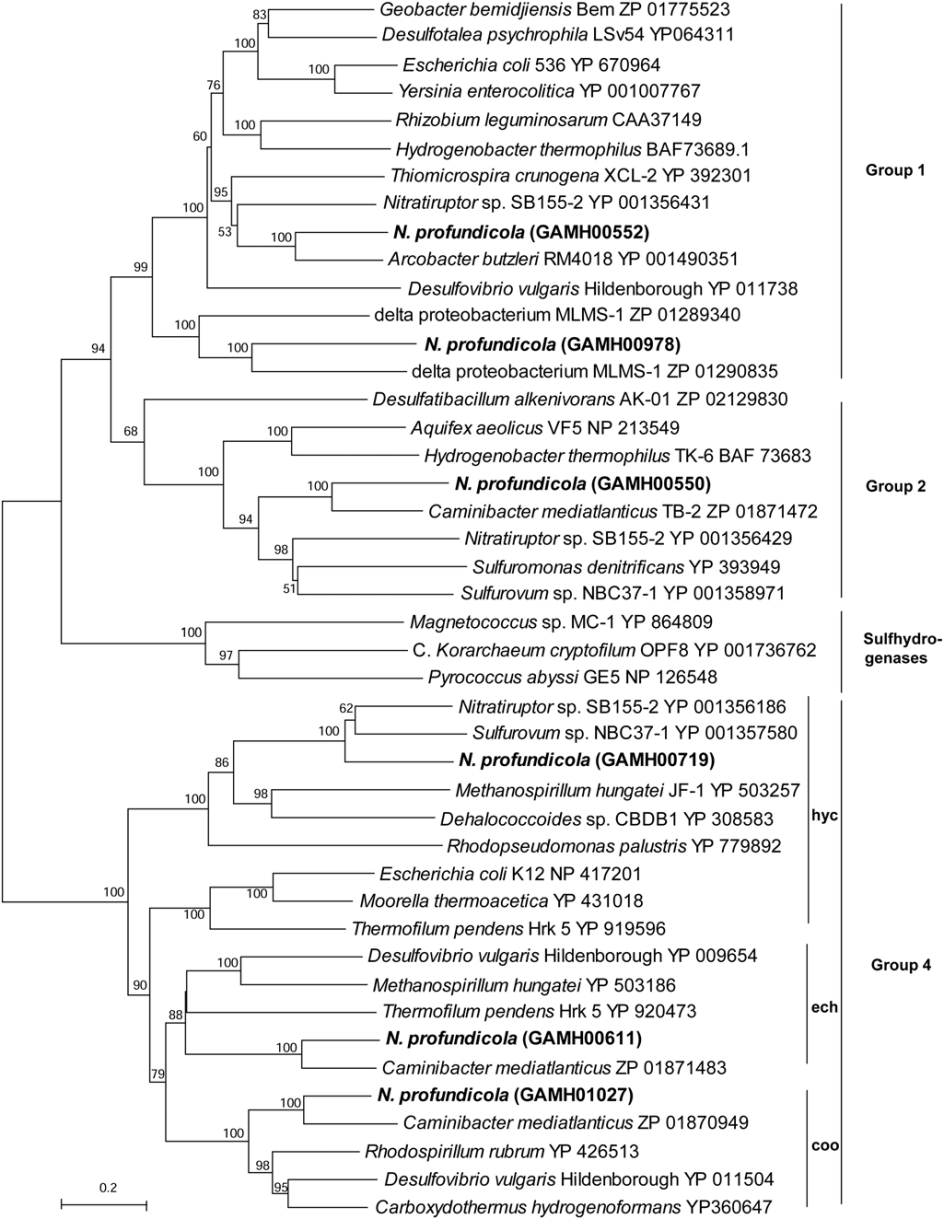
Phylogenetic relationships of large subunit hydrogenase protein sequences. Distance topologies were performed in MEGA4, based on the Neighbor-joining method after alignment with ClustalW (BLOSUM matrix). Bootstrap values (500 replicates) are indicated on branches. Indicated groupings are based on previous analyses.

*N. profundicola* has a number of conserved genes involved in energy metabolism. We concentrate here on those that are unique, either to *N. profundicola* or other hydrothermal vent microbes. The importance of H_2_ metabolism observed in the physiology of *N. profundicola* is clearly reflected by the number of operons for hydrogenases. As evidenced by the phylogenetic relationships of the large subunits, there are one H_2_-sensing (Group 2), two H_2_-uptake (Group 1), and three H_2_-evolving (Group 4) hydrogenases in the *N. profundicola* genome (Figure 5). One of the membrane uptake (GAmH_0551-0556) and the sensing (GAmH_0548, 0549) hydrogenase operons are similar to those in other vent *Epsilonproteobacteria* (*Nitratiruptor sp.*, *Sulfurovum*, *C. mediatlanticus*). The second uptake hydrogenase (NiFeSe, GAmH_0978, GAmH_0979) is most similar to that from *Desulfotalea psychrophila* LSv54 (DP0159, DP0160), *Deltaproteobacterium* MLMS-1, and other members of the *Deltaproteobacteria*, and the gene product is likely soluble and periplasmic because there is no membrane anchor subunit. It is induced in *Desulfovibrio vulgaris str.* Hildenborough under H_2_- and Se-replete conditions [88]. There are three predicted operons in the *N. profundicola* genome for H_2_-evolving hydrogenases (Group 4): *ech*, *hyc*, and *coo*. The *ech* (GAmH_0607-0612) and *hyc* (GAmH_0723-0718) operons are identical in gene order to those in other described genomes (e.g., *ech*, *Desulfovibrio vulgaris* and *Methanosarcina barkeri*; *hyc*, *Rhodopseudomonas palustris* and, *Methanosarcina mazei*) with two internal genes conserved in the *hyc* operon not found in the *E. coli hyc* operon [89],[90],[91],[92]. The *ech* genes are most similar to that of *C. mediatlanticus*. Although both *N. profundicola* and *C. mediatlanticus* have the large and small subunits of CO-induced hydrogenase (CooLH), only *N. profundicola* contains the full operon structure (cooMKLXUHF3, GAmH_1032-1026), similar to both *Desulfovibrio vulgaris* and *Carboxydothermus hydrogenoformans*, (Figure S2) [89],[93].

The physiological roles of the multiple hydrogenases likely reflect an adaptation to variable hydrogen and electron acceptor concentrations, though defining their specific roles will require significant experimental effort. We propose that the Group 1 membrane bound hydrogenase is a constitutive system that couples to the quinone pool and contributes to establishing and maintaining the proton gradient across the cytoplasmic membrane. Three of the hydrogenases appear to couple with ferredoxin. The reduction of ferredoxin with molecular hydrogen is thermodynamically unfavorable due to the difference in redox potentials with the reaction becoming even more unfavorable at low hydrogen concentrations. Two of the Group 4 energy-conserving hydrogenases may be able to utilize proton or sodium motive force to overcome this energy requirement and may be utilized to help provide reduced ferredoxin for CO_2_ fixation when hydrogen concentrations are low. This has been proposed for the homologous *Methanosarcina barkeri* Ech hydrogenase during autotrophic growth [94]. The soluble, cytoplasmic Group 2 hydrogenase cannot couple to ionic gradients, and we therefore hypothesize that it may only be important when hydrogen concentrations in the environment are high and the cell rapidly oxidizes reduced ferredoxin, conditions that would minimize the unfavorable energetics. This scenario has been proposed for the *Aquifex aeolicus* cytoplasmic hydrogenase, the closest characterized homolog to that of *N. profundicola* [31],[95]. Alternatively, this enzyme could be important during hydrogen and polysulfide starvation. Under these conditions, strain AmH may degrade internal carbon stores by running the rTCA cycle oxidatively, and the hydrogenase would reoxidize reduced ferredoxin, allowing the cycle to continue and produce hydrogen in a favorable reaction. The aforementioned Group 4 hydrogenases could also contribute in this scenario by maintaining a proton or sodium gradient. The role of the third Group 4 hydrogenase, CooLH, is enigmatic as *N. profundicola* does not, nor do any of the other *Epsilonproteobacteria*, encode a CooS-type CODH subunit that is required for CO-dependent H_2_ evolution in *Rhodospirillum rubrum* [96]. Similarly, the role of the periplasmic Group 1 hydrogenase is unclear.

Besides the hydrogenases and a typical F_1_F_0_ ATPase enzyme complex (F_0_, GAmH_1440; F_1_, GAmH_0301-0306) closely related to other *Epsilonproteobacteria* ATPases, a vacuolar-type H^+^-transporting ATPase operon (GAmH_0030-0023) was also found in the *N. profundicola* genome. The genes in the operon are most closely related to *Halothermothrix orenii* H168 and *Clostridium tetani* E88, and other members of the *Clostridia* (*Firmicutes*) [97],[98]. Similar operon structures are found in *Clostridium tetani* and *Nitrosococcus oceani* and differ somewhat from the archaeal operons (e.g., *Methanosarcina mazei*, *Pyrococcus horikoshi*, and *Thermococcus kodakaraensis* KOD1) [87],[92],[97],[99]. Since this operon is only in the *N. profundicola* and not in any other epsilonproteobacterial genome, it was likely acquired via a horizontal gene transfer event. The function of this complex may be partially similar to that of *N. oceani* and other AOB, where it actively extrudes sodium [100]. However, *N. profundicola* lacks sodium-dependent complex I that in *N. oceani* likely reduces NAD to NADH [87]. *N. profundicola* contains several types of homologous genes that code for sodium transport proteins: sodium-proton antiporters (GAmH_0108, GAmH_1552, GAmH_0762, GAmH_0955) most likely used for motility and transport processes; a putative sodium-proline symporter (GAmH_0965, PutP) for amino acid uptake; and a putative sodium di- and tricarboxylate transporter (GAmH_0459, TrkA-C). *N. profundicola* likely uses the V-type ATPase to regulate the influx of sodium ions from these processes.

The *N. profundicola* genome contains all but 4 (*nuo*E, F, G, K) of the subunits for NADH:ubiquinone oxidoreductase (NADH dehydrogenase, complex I), which in other species contributes to proton motive force generation for oxidative phosphorylation. The gene structure here is identical to some other anaerobic bacteria, including *C. mediatlanticus* and *Carboxydothermus hydrogenoformans*, but is missing two genes (F and G) that normally form the inner cytoplasmic structure of the oxidoreductase found in facultative anaerobes and microaerophilic bacteria belonging to the *Epsilonproteobacteria*. The missing part makes up the acceptor region of the molecule, and therefore these anaerobes most likely have a different acceptor portion of the complex not found in the *nuo* operon. In addition to the fumarate reductase mentioned above, the genome also contains a four-subunit formate dehydrogenase (GAmH_1274-1277), which enables *N. profundicola* to grow with formate as an electron donor and carbon source [21].

**Figure 6:**
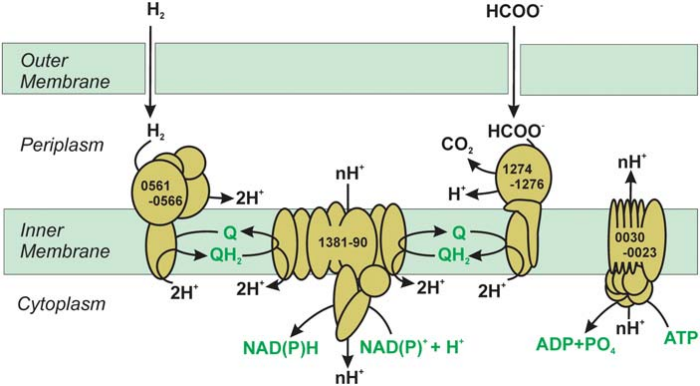
Generation of NAD(P)H in *N. profundicola* via the predicted partial NUO complex from hydrogen or formate. The ultimate reduction of NAD(P)+ from the quinone pool is endergonic and requires energy in the form of coupling to the proton motive force, which may be provided, in part, by a V-type ATPase. Numbers on complexes refer to the ORF numbers of the encoding genes.

Cell energy requirements include both the reduced ferredoxin to drive rTCA cycle CO_2_-fixing reactions and reduced pyridine nucleotides to drive other anabolic processes. The production of reduced ferredoxin from hydrogen was discussed above. As *N. profundicola* lacks the genes necessary to generate NAD(P)H via the Pentose Phosphate pathway or via a ferredoxin-linked NADH oxidoreductase, we hypothesize that the partial complex I (NUO) acting in concert with formate dehydrogenase (FDH) or the membrane-bound uptake hydrogenase could form a reverse electron transport system responsible for producing NAD(P)H (Figure 6). Additional proton motive force to drive the system could be supplied by the V-type ATPase. In addition to hydrogen and formate, the polysulfide reductase might be able to act in the reverse direction and oxidize sulfide to polysulfide, or succinate could be oxidized to fumarate via a succinate dehydrogenase. Both of these activities would also reduce the quinone pool to provide electrons under low hydrogen/high sulfide, and/or inorganic electron donor-limited conditions. This flexibility in electron donor usage may be an adaptation of *N. profundicola* to the highly variable hydrothermal vent environment.

### DNA Replication and Repair

Several DNA replication genes found in all vent *Epsilonproteobacteria* (but not exclusive to this group) include: nucleotidyltransferase/DNA polymerase (GAmH_0589); DNA polymerase (Pol) III epsilon subunit and related 3′–5′ exonucleases (GAmH_0148, GAmH_0193); and the gram positive type of DNA Pol III alpha subunit (GAmH_0988, GAmH_1246), which contains an internal proofreading exonuclease [101]. Only four *Epsilonproteobacteria*, including *N. profundicola* and *C. mediatlanticus*, contain multiple homologs of the Gram-positive DNA Pol III alpha subunit, and most of the vent *Epsilonproteobacteria* and a few free-living members contain 2–3 homologs of the epsilon subunit. The epsilon subunit provides proofreading for DNA Pol III (*mutD* in *E. coli*), and binds to the polymerase and histidinol phosphatase (php) domain of the *E. coli* DNA Pol III alpha subunit [102]. Wieczorek et al. (2006) suggested that the similar php domain found in DNA PolX, mentioned below, might also bind the epsilon subunit, enabling an additional coupled proofreading reaction beyond the DNA Pol III enzyme complex. The inventory of epsilon and alpha DNA Pol III subunits and DNA Pol X in vent *Epsilonproteobacteria* supports this hypothesis.

There are three genes whose functions relate to DNA replication and repair and are found exclusively in the vent *Epsilonproteobacteria* and not in the subdivision as a whole; they may be very important in the repair of damaged DNA required under the extreme conditions found at vents (Table 1). First, a homolog to the DNA-repair photolyase or spore photoproduct lyase gene (GAmH_0507) is present. It is a member of the radical S-adenosylmethionine (SAM, GAmH_0452 in *N. profundicola*) domain protein superfamily (Figure S3). This gene shares a strong similarity to other members of this superfamily cluster found in three out of four of the vent *Epsilonproteobacteria* (data not shown) but is not found in non-vent Epsilonproteobacteria. The most similar experimentally characterized protein to the gene in *N. profundicola* is a gene from *Bacillus subtilis* (23% protein sequence identity) which functions to repair thymine dimers (5-thyminyl-5,6-dihydrothymine, or spore product) specifically formed in spores during UV exposure [103]. Although there is evidence of some light emissions from deep-sea hydrothermal vents, most likely from geothermal energy found above 700 nm [104], there is no evidence of UV emissions. A member of this family was also identified in the genome of *Idiomarina loihiensis* L2TR, a *Gammaproteobacterium* isolated from a deep-sea hydrothermal vent at Loihi, Hawaii [105],[106]. In these vent bacteria, the related gene product could function in repair of damage to DNA generated by other processes. Possible conditions that could lead to nucleotide damage include high levels of ionizing radiation and metal complexes with reactive oxidative species [8], [107]–109. While there are few direct measurements of ionizing radiation on chimney surfaces, researchers have isolated bacteria and archaea resistant to high levels of radiation (>20–30 kGy) and found polychaetes containing very high levels of natural Pb-210 and Po-210 [5]–[8].

The second gene predicted to be involved in DNA replication and repair found in all vent *Epsilonproteobacteria* is DNA polymerase IV, family X (PolX, GAmH_1417). PolX homologs are found in 25–30% of sequenced bacterial genomes, and genetic evidence indicates that PolX contributes to double strand break repair in the radiation-resistant bacterium *Deinococcus radiodurans* [110]. Eukaryotic PolX homologs act to repair double strand breaks with 3′-overhangs containing gaps on both strands, i.e., non-homologous end repair. While experimental and bioinformatics evidence suggest that a Ku-LigD system for non-homologous end repair is present in a wide variety of bacterial groups, it does not appear to be present in *Epsilonproteobacteria* [111],[112]. Therefore, PolX in *Epsilonproteobacteria* may substitute for the Ku-LigD system in countering DNA damage.

**Figure 7:**
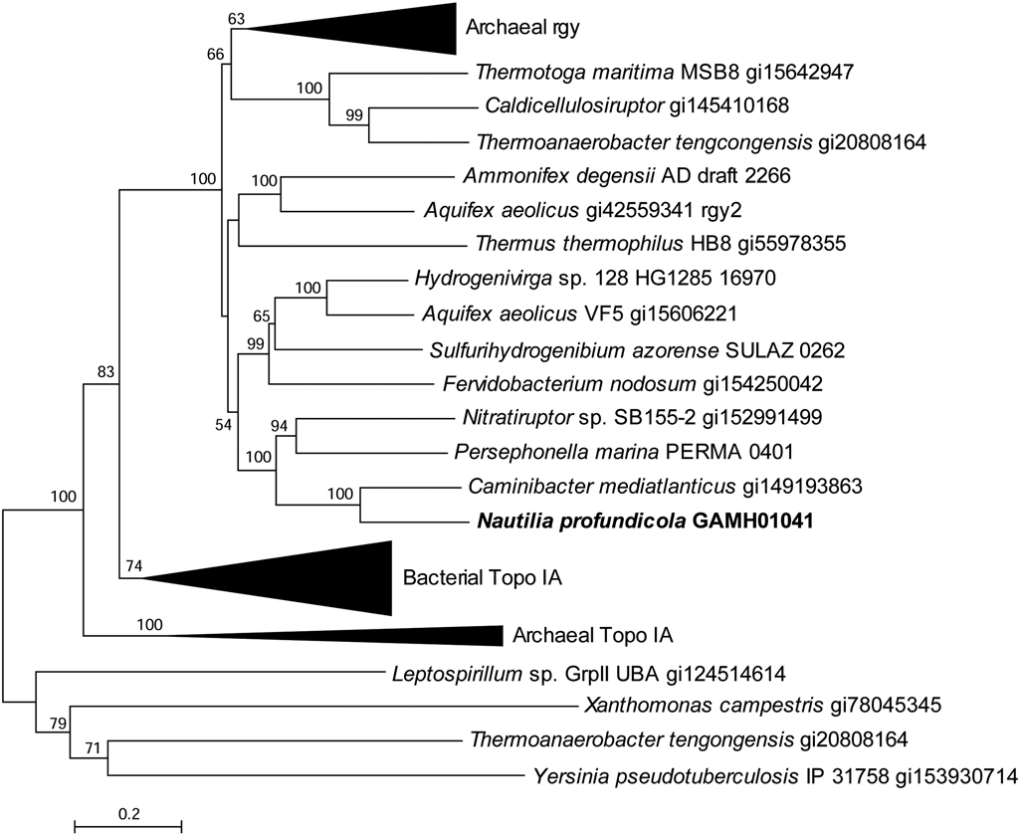
Phylogenetic relationships between reverse gyrase and related topoisomerase protein sequences. Distance topologies were performed in MEGA4, based on the Neighbor-joining method after alignment with ClustalW (BLOSUM matrix). Bootstrap values (500 replicates) are indicated on branches. Nearly identical topologies were observed with Maximum Likelihood (phyml), Minimum Evolution and Maximum Parsimony algorithms (data not shown).

Finally, a gene involved in DNA stability at high temperatures was found exclusively in vent associated *Epsilonproteobacteria*: reverse gyrase (*rgy*, GAmH_1041, Figure 7). Rgy, long considered a hallmark protein of hyperthermophiles, likely acts as a DNA chaperone by preferentially binding to nicked DNA during repair [113]. Reverse gyrase is named for its ATP-dependent introduction of positive supercoiling, which protects against DNA damage at high temperatures and may also introduce negative supercoiling [114]–[116]. Reverse gyrase has previously been reported only in microbes whose optimal growth temperatures are above 65°C, over 20°C above that of *N. profundicola* [117]. Although considered a hallmark protein of hyperthermophiles, Rgy may not be essential for growth at high temperatures. A *rgy* mutant of *Thermococcus kodakaraensis* KOD1 grew at high temperatures, albeit more slowly than the wild type, with a corresponding decrease in the upper growth limit [118].

We report here that other deeply-branching hydrothermal vent *Epsilonproteobacteria*, *Caminibacter mediatlanticus* [19] and *Nitratiruptor sp.* SB155-2 [18], also contain a highly related *rgy* sequence; their optimal growth temperatures are 55°C (Table 1, Figure 7). Brochier-Armanet and Forterre (2006) argued for an ancient bacterial acquisition of *rgy* from Archaea. However, the analysis presented here indicates a clear separation between the bacterial and archaeal clades, which suggests descent from a common ancestor. *Thermotoga* and *Clostridia rgy* sequences do form a separate clade closer to the archaeal clade than other bacterial groups (Figure 7), perhaps suggesting a lateral transfer in some groups. The *rgy* gene is not found in the genome of one sequenced hydrothermal vent member of the *Epsilonproteobacteria*, *Sulfurovum* sp. NBC37-1 [18]. This strain, which is mesophilic (T_opt_ = 33°C), was isolated from an in situ sampler and is phenotypically more similar to *Thiomicrospira crunogena*, a vent- associated microaerobic sulfur-oxidizing chemoautotroph that also does not contain the *rgy* gene [27],[29].

**Figure 8:**
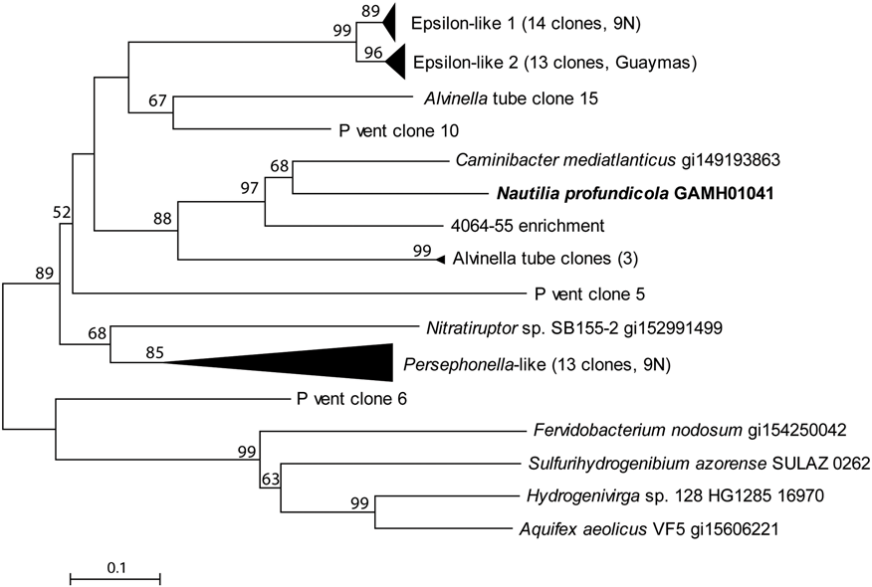
Phylogenetic relationships between bacterial reverse gyrase sequences obtained from PCR amplification from hydrothermal vent chimneys and known sequences. Distance topologies were performed in MEGA4, based on the Neighbor-joining method after alignment with ClustalW (BLOSUM matrix). Bootstrap values (500 replicates) are indicated on branches.

A PCR survey of thermophilic *Nautiliales* from geographically distinct sites indicated that an *rgy* gene is also present in *Lebetimonas acidiphila* (T_opt_ = 50°C) [25] and *Nautilia sp.* strain 4064-55 (T_opt_ = 55°C). These sequences formed a monophyletic clade with *N. profundicola* and *C. mediatlanticus rgy* sequences (Figure 8). However, *Nautilia lithotrophica*, isolated from the same geographic location as *N. profundicola* [23], returned negative results in this survey. These results suggest that if the *Epsilonproteobacteria* acquired *rgy* by horizontal gene transfer, it would have been prior to their split from other bacterial groups or prior to the split of the *Nautiliales* order, whereby some could have lost this gene or the gene sequence diverged enough to be undetectable with the degenerate primers used in the study.

**Figure 9:**
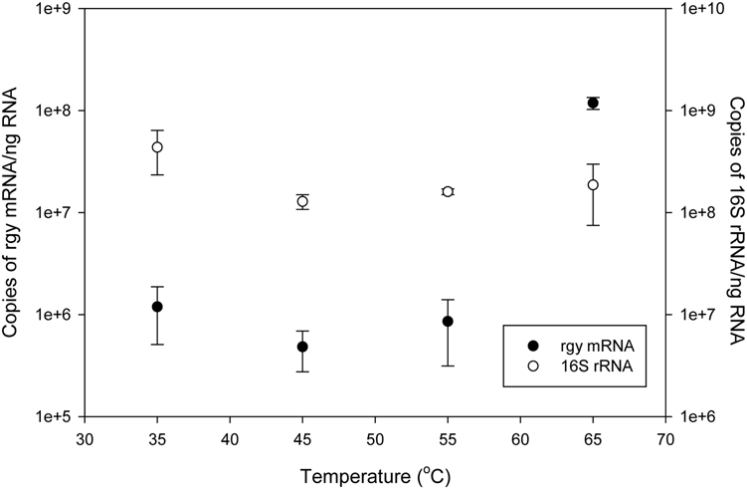
Abundance of *rgy* mRNA and 16S rRNA in *N. profundicola* cells incubated under the indicated temperature for 2 hours. Values were calculated per ng of RNA. Standard errors indicated by the error bars. Y axes are on a log scale.

To determine if *rgy* might be involved in stress response or DNA repair, expression of this gene in cultured cells was monitored by quantitative reverse transcription-PCR (qRT-PCR). Transcript abundance of *N. profundicola rgy* increased over 100 fold when actively growing cells were exposed to 65°C while rRNA transcript abundance remained constant or slightly decreased (Figure 9). The same response was not observed at 35, 45, or 55°C. These results indicate that *N. profundicola rgy* transcription is induced by thermal stress and, by extension, suggest that the *N. profundicola rgy* gene product is involved in at least thermal stress response. Our results agree with previous data suggesting that Rgy activity is increased in hyperthermophiles at higher temperatures [114]. The upregulation of *rgy* at higher temperatures, combined with previous data that suggest that Rgy is not absolutely required for growth at high temperatures [118], may imply that Rgy is involved in other functions, such as gene regulation or promoter activity.

**Figure 10:**
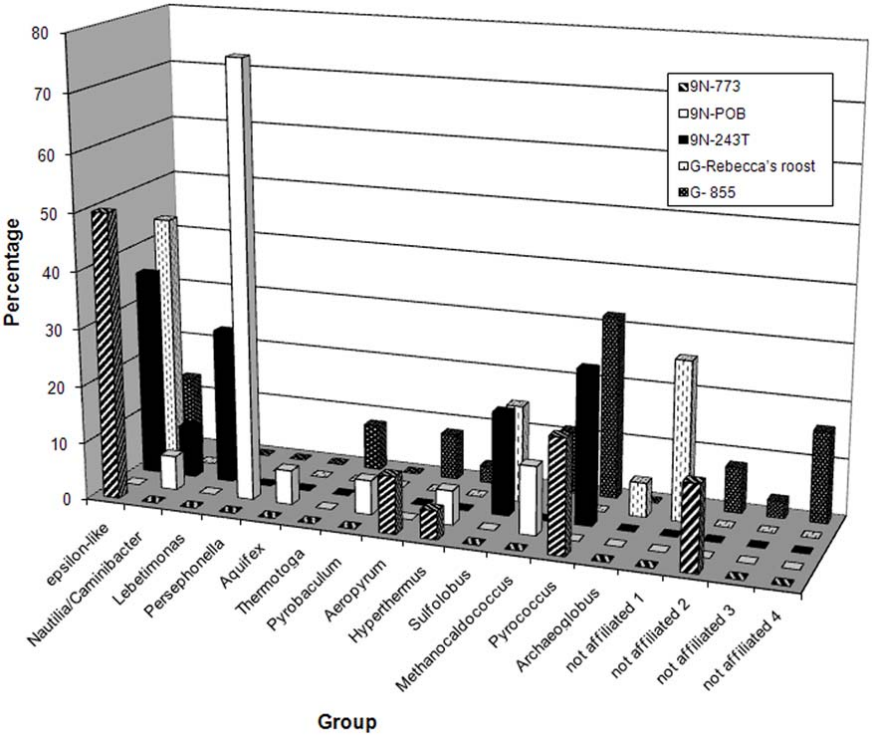
Phylogenetic associations and relative percentages of *rgy* clones retrieved from hydrothermal vent chimney samples. 9N-773 (Io vent); 9N-POB (P vent, bottom); 9N-243T (Alvinella tube scraping); G-Rebecca's Roost (Guaymas Basin flange); G-855 (Guaymas Basin flange). Groups were reported as clades with nearest cultured representatives because of poor bootstrap values on the distal branch points, most likely due to the length of the aligned sequence.

To further extend these observations, the occurrence, phylogeny, and expression of *rgy in vivo* was assessed in representative deep-sea hydrothermal vent samples from 9°N and Guaymas Basin along the East Pacific Rise. PCR with degenerate primers produced a number of *rgy* sequences from these samples, including two groups (referred to as Epsilon-like) that are distinct from *rgy* sequences present in cultured members of the *Epsilonproteobacteria* (Figures 8, 10). These Epsilon-like *rgy* sequences were the most frequently observed in the vent survey with one exception. Primers specific for the geographically distinct members of the Epsilon-like groups were used in qPCR experiments on samples from varying temperatures. These experiments indicated that the Epsilon-like groups were more abundant in samples from chimneys with lower temperatures (8–20°C), where as much as 27% of the bacterial population, when normalized to 16S rRNA gene abundance, appeared to contain an Epsilon-like *rgy* gene (Table 3). Perhaps organisms containing the Epsilon-like *rgy* gene may normally experience low temperatures, but display enhanced resistance to short term temperature spikes. In support of this theory, RT-PCR with specific Epsilon-like *rgy* primers demonstrated that this gene is transcribed *in situ* (data not shown).

**Table 3:** Abundance of epsilonproteobacterial-like *rgy* genes in hydrothermal vent chimney samples, normalized to 2.5 copies of all bacterial 16S rRNA genes.

*Chimney*	*Temperature (°C)*	*% of bacteria with Epsilon-like* rgy
*9°N*		
Io vent	48	11.3 (0.32)
M vent	310^1^	4.3 ( 0.24)
M vent	>225^1^	0.7 (0.33)
Bio vent	>350^1^	1.5 (0.30)
*Guaymas Basin*		
Rebecca's roost flange	60–70	3.0 (0.33)
Robin's Roost	15–52	5.4 (0.17)
Guaymas flange	8–20	27.1 (0.32)

1 Temperatures taken in actively venting black smoker fluid.

Propagated standard errors are shown in parentheses.

### Conclusions

The *Epsilonproteobacteria*, while perhaps better recognized as pathogens, are supremely adapted to hydrothermal vent ecosystems [11]. The *N. profundicola* genome reported here indicates that this organism's success in the vent environment relies on chemoautotrophy via the rTCA cycle and a tendency to reduce sulfur species driven by a prodigious capacity for hydrogen oxidation. It is truly remarkable that *N. profundicola* lacks much of the well-established electron transport chain inventory, but is able to utilize its large suite of hydrogenases and ATPases, the most described yet in any *Epsilonproteobacteria*, for energy and reducing equivalents. Presumably, these genes and pathways would be tightly regulated to follow the prevailing redox conditions found at highly fluctuating diffuse flow vents. The abundance of signal transduction genes similar to other free-living vent *Epsilonproteobacteria* implies that the lifestyle of *N. profundicola* is mainly independent of *A. pompejana* and its episymbionts. The *N. profundicola* genome also produced two unique insights that will drive further investigations. First, we propose a novel pathway for nitrate assimilation where Nap, HURM (Hao and *c*_M_552), and Har function together to produce ammonium via the toxic intermediate hydroxylamine.

Second, *N. profundicola*, along with *C. mediatlanticus* and *Nitratiruptor* sp., contains a gene homologous to reverse gyrase, which most likely confers a selective advantage to these *Epsilonproteobacteria* in the face of rapid temperature fluctuations that occur at deep-sea hydrothermal vents. *N. profundicola*, therefore, contains all the genes necessary for life in conditions widely believed to reflect those in the archaean biosphere: anaerobic, sulfur, H_2_- and CO_2_-rich, with unstable redox potentials and temperatures [119],[120]. *N. profundicola* will thus be an important model system to understand early microbial life on earth.

## Material and Methods

### Growth and Sequencing of *N. profundicola*

*N. profundicola* (*Nautilia sp.* AmH) was grown in an anaerobic autotrophic sulfur medium as described [20]. DNA was extracted from the cultured cells using a standard SDS/proteinase K/phenol/chloroform extraction technique [121]. The genome was sequenced with the whole-genome shotgun method with both small and medium insert libraries as previously described [122]. Physical and sequencing gaps were closed as described [122]. Assembly was performed with the Celera Assembler version 3. An initial set of predicted ORFs and the functional assignment of genes was performed using the TIGR (now JCVI) autoannotation pipeline. This included gene finding with Glimmer, Blast-extend-repraze (BER) searches, HMM searches, TMHMM searches, SignalP predictions, and automatic annotations from AutoAnnotate.

*For nitrate growth analysis*, *N. profundicola* was cultivated in anaerobic salt water media with S^0^ and a headspace of H_2_/CO_2_ gas as previously described [21], with either 5 mM ammonium or 20 mM nitrate as the sole nitrogen source. After three passes in the indicated media, cell growth was monitored by direct counts of cells stained with 4′, 6-diamidino-2-phenylindole using an Olympus Provis AX70 microscope and image analysis software (ImagePro Plus, Media Cybernetics). The production of hydrogen sulfide was monitored during cell growth using the Cline method [123].

### Annotation

The manual annotation tool Manatee was used to manually review the output from the prokaryotic pipeline of the TIGR (now JCVI) annotation pipeline (http://manatee.sourceforge.net/). Manual curation was performed on approximately 383 CDS. Further refinements and comparisons to other genomes were done with IMG, BLAST, and RAST [124]–[127]. GenBank accession number of the genome is CP001279. The *N. profundicola* genome report as recommended by Genome Standards Consortium [128] is outlined (Table S1).

To identify redundant/duplicated operons, an all-vs-all self blastp was performed and an E-value cutoff (of 10–20) was applied to the results to establish links between proteins. The results were then visually analyzed using DagChainer [129], and no evidence of duplicated operons was found. Potential prophage regions were located by manually searching genome regions near tRNA as well as with the automated online tool, Prophage Finder [38], which looks for phage-like genes and changes in G+C content.

### Phylogenetic Analyses

Phylogenetic trees of the translated *rgy* and large subunit hydrogenase sequences were constructed in MEGA4 [130] with the default parameters, based on both Neighbor-joining and minimum evolutionary algorithms with 500 bootstrap replicates. PHYML trees were calculated with the following parameters: Bootstrap replica number: 100; Substitution model: JTT; Proportion of invariable sites: automatic estimated by the program; Number of substitution rate categories: 4; Gamma distribution parameter: automatic estimated by the program; and Starting tree: BIONJ. The program was allowed to optimize the topology, the branch lengths and rate parameters of the start tree.

### Abundance of *N. profundicola rgy* mRNA

Cells were grown at 45°C to mid-log phase (∼12 hours) and triplicate tubes shifted to each indicated temperature for 2 hours. RNA was harvested using a CTAB extraction protocol [131], and genomic DNA was removed by gDNA wipeout buffer (Qiagen QuantiTect Reverse Transcription Kit). RNA samples were quantified using the RiboGreen Assay (Invitrogen) and diluted to the same concentrations with RNAase-free H_2_O. cDNA was produced using random primers and the Qiagen QuantiTect Reverse Transcription Kit.

To estimate the abundance of *rgy* mRNA, specific primers were designed from the nucleotide sequences of the *N. profundicola rgy* gene (AmHrgy2022F: 5′-CACAGACCCCGA TAGGGAAGGTG; AmHrgy2203R: 5′-ACCCGACCCATCTGTCCGTAATTC). In addition, the 16S rRNA was quantified using primers and conditions described elsewhere [132],[133]. Genomic DNA was quantified using the picogreen assay (Invitrogen) and used in standard curves for both reactions. Quantitative PCR was performed in triplicate or quadruplicate with 1 µl of cDNA in a final volume of 12.5 µl using the Stratagene SYBR green mix on an ABI7500, and PCR conditions for the *rgy* primer set were: 95°C 10 minutes; followed by 40 cycles of amplification at 95°C for 15 seconds, 62°C for 30 seconds and 72°C for 45 seconds, with a final dissociation step. Final *rgy* primer concentrations were at 0.05 µM.

### Environmental Sample Analyses

Chimney and *Alvinella pompejana* tube samples were collected as described previously during four cruises to the EPR in November 1999 (9°N, Io vent -773), January 2000 (Guaymas Basin, 534G and 855G), the Extreme cruises Extreme 2001, November 2001 (9°N, P vent-POB) and Extreme 2003, November 2003 (*Alvinella pompejana* tube-243T). In addition, a chimney sample (Michael's vent, Extreme 2004 cruise, Nov.-Dec., 2004, 9°N, EPR) was inoculated into anaerobic autotrophic sulfur medium as described [20] and incubated at 55°C. One strain (*Nautilia sp.* strain 4064-55) was detected in the third subculture by DGGE and sequenced as described [20]. DNA and RNA were extracted from the indicated samples and RNA was reverse transcribed as described previously with a modified *rgy* reverse PCR primer, without the KpnI restriction site [20],[134].

### Reverse Gyrase PCR Amplifications, Cloning, and Abundance Estimates

A portion of the *rgy* gene from environmental samples was PCR amplified with degenerate primers as previously described [134], cloned into a TOPO TA vector, and sequenced [26]. GenBank accession numbers of the clones are FJ597068-FJ597141. Two *rgy* clades (Epsilon-like 1 and Epsilon-like 2) were chosen for quantitative estimates based on SYBR green quantification on an ABI7500. Primers were: Egrp1 rgyF (5′-TGGATCGGTTTCGGAATTTCCG); Egrp1 rgyR (5′-GCAAATGCGTTCTCGAAACGTTC); Egrp2 rgyF (5′-GGTGGATCGGATTCGGGATTTC); and Egrp2 rgyR (5′-CTTGAATGCGAACTCGAGGCG). Conditions and primer concentrations were as described for RT-qPCR above, but with a two-step PCR reaction (95°C for 15 seconds, 65°C for 2 minutes). Standard curves were generated from dilutions of cloned DNAs within the specific clade (Epsilon-like 1 or 2).

## Supporting Information

Figure S1 Alignment of the C-termini of Hao proteins with annotations based on Igarashi et al. [135] and Bergmann et al. [136]: CxxCH, heme binding motif 8; H459, axial ligand to heme; Y467, protein crosslink to catalytic heme; major alpha helices; alpha TMS, C-terminal transmembrane spanning domain. Sequence sources: CAD84873, *Nitrosomonas europaea* ATCC 19718; ABB75723, *Nitrosospira multiformis* ATCC 25196; ABA57404, *Nitrosococcus oceani* ATCC 19707; AAU92745, *Methylococcus capsulatus* Bath; EDM23995, *Caminibacter mediatlanticus* TB-2; AM-H, *Nautilia profundicola* AM-H. [135], [136].
(4.15 MB TIF)

Figure S2 Gene neighborhood of rgy gene (Reverse gyrase, GAMH_1041), coo (CO-hydrogenase, GAMH_1032-1026) and fdr (Fumarate reductase, GAMH_1024-1022) operons in *Nautilia profundicola* (A) and *Caminibacter mediatlanticus* (B). Genes of the same color (except light yellow) are from the same orthologous group (top COG hit).
(2.94 MB TIF)

Figure S3 Gene neighborhoods of the spl-related gene (DNA photolyase, Radical SAM domain protein) in *Nautilia profundicola* (A) and *Caminibacter mediatlanticus* (B). Genes of the same color (except light yellow) are from the same orthologous group (top COG hit). Abbreviations: spl = DNA photolyase; H = hypothetical protein; CH = conserved hypothetical; 1 = GAMH_0510, xanthine guanine phosphoribosyl transferase; 2 = GAMH_0506, short chain dehydrogenase; 3 = GAMH_0503, plasmid stabilization system; 4/5 = GAMH_0504/502, toxin/antitoxin gene pair, RelBE-3 family. Arrows indicate a probable genome inversion between the strains.
(7.78 MB TIF)

Table S1 *Nautilia profundicola* genome sequence report as recommended by the Genome Standards Consortium [137].
(0.06 MB DOC)
