# Supplementary figures and images for: Adaptations to Submarine Hydrothermal Environments Exemplified by the Genome of Nautilia profundicola
Source: PLoS Genet. 2009 Feb 6;5(2):e1000362. doi: 10.1371/journal.pgen.1000362 (PMC2628731; doi:10.1371/journal.pgen.1000362)

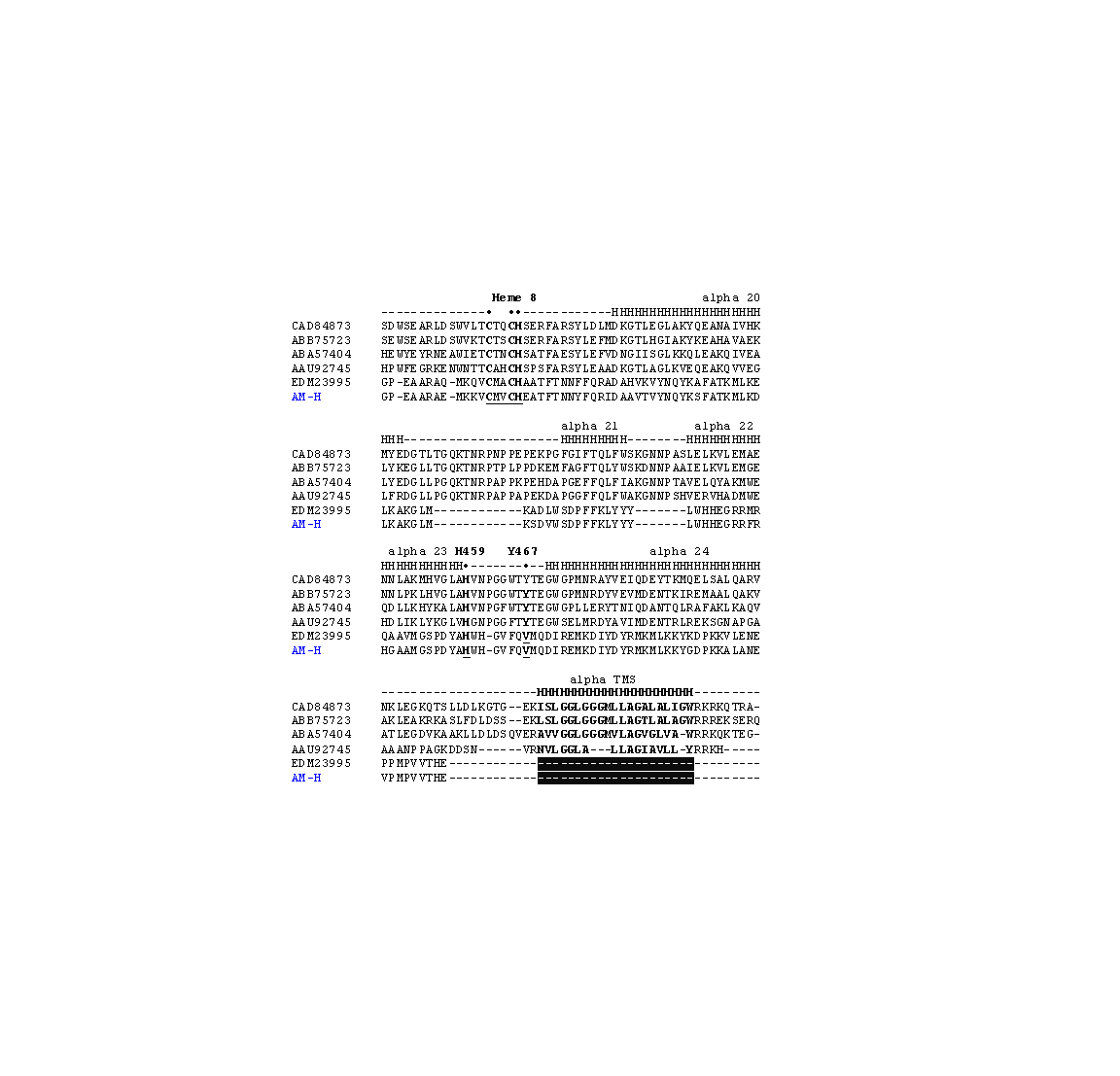

Supplement: Figure S1 — Alignment of the C-termini of Hao proteins with annotations based on Igarashi et al. [135] and Bergmann et al. [136]: CxxCH, heme binding motif 8; H459, axial ligand to heme; Y467, protein crosslink to catalytic heme; major alpha helices; alpha TMS, C-terminal transmembrane spanning domain. Sequence sources: CAD84873, Nitrosomonas europaea ATCC 19718; ABB75723, Nitrosospira multiformis ATCC 25196; ABA57404, Nitrosococcus oceani ATCC 19707; AAU92745, Methylococcus capsulatus Bath; EDM23995, Caminibacter mediatlanticus TB-2; AM-H, Nautilia profundicola AM-H. [135], [136]. (4.15 MB TIF) [file pgen.1000362.s001.tif]

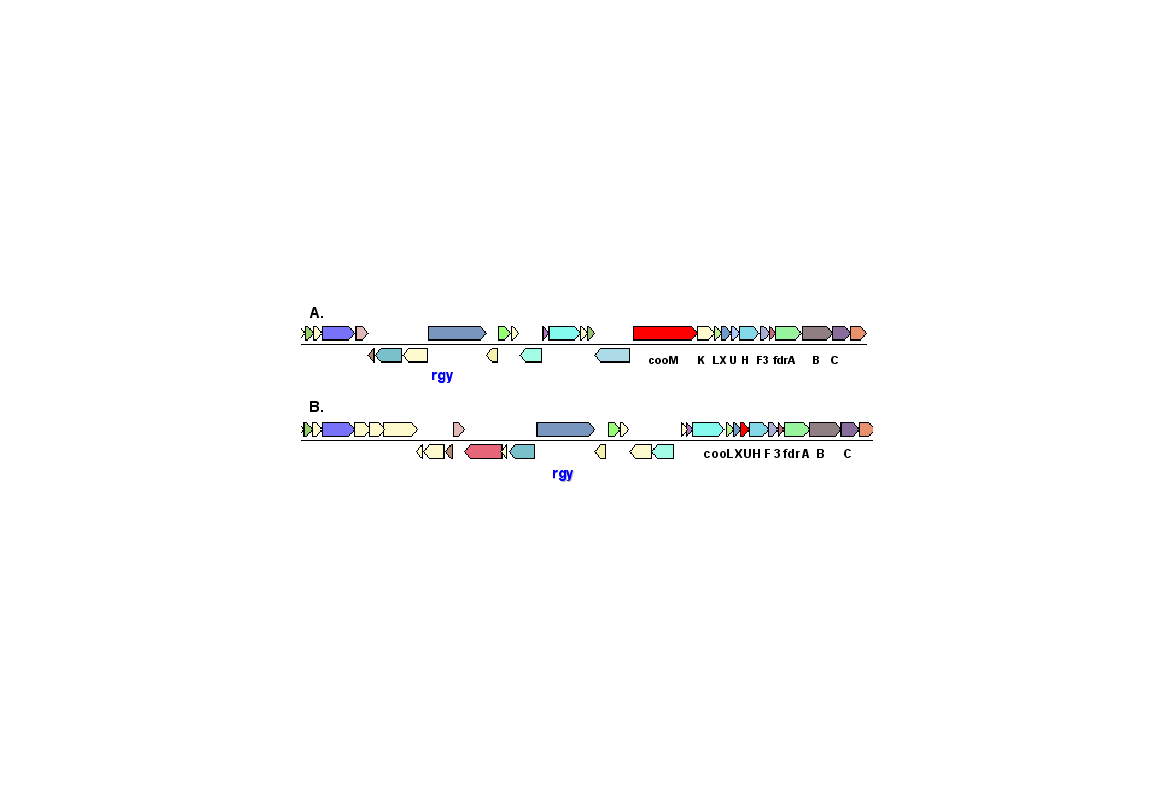

Supplement: Figure S2 — Gene neighborhood of rgy gene (Reverse gyrase, GAMH_1041), coo (CO-hydrogenase, GAMH_1032-1026) and fdr (Fumarate reductase, GAMH_1024-1022) operons in Nautilia profundicola (A) and Caminibacter mediatlanticus (B). Genes of the same color (except light yellow) are from the same orthologous group (top COG hit). (2.94 MB TIF) [file pgen.1000362.s002.tif]

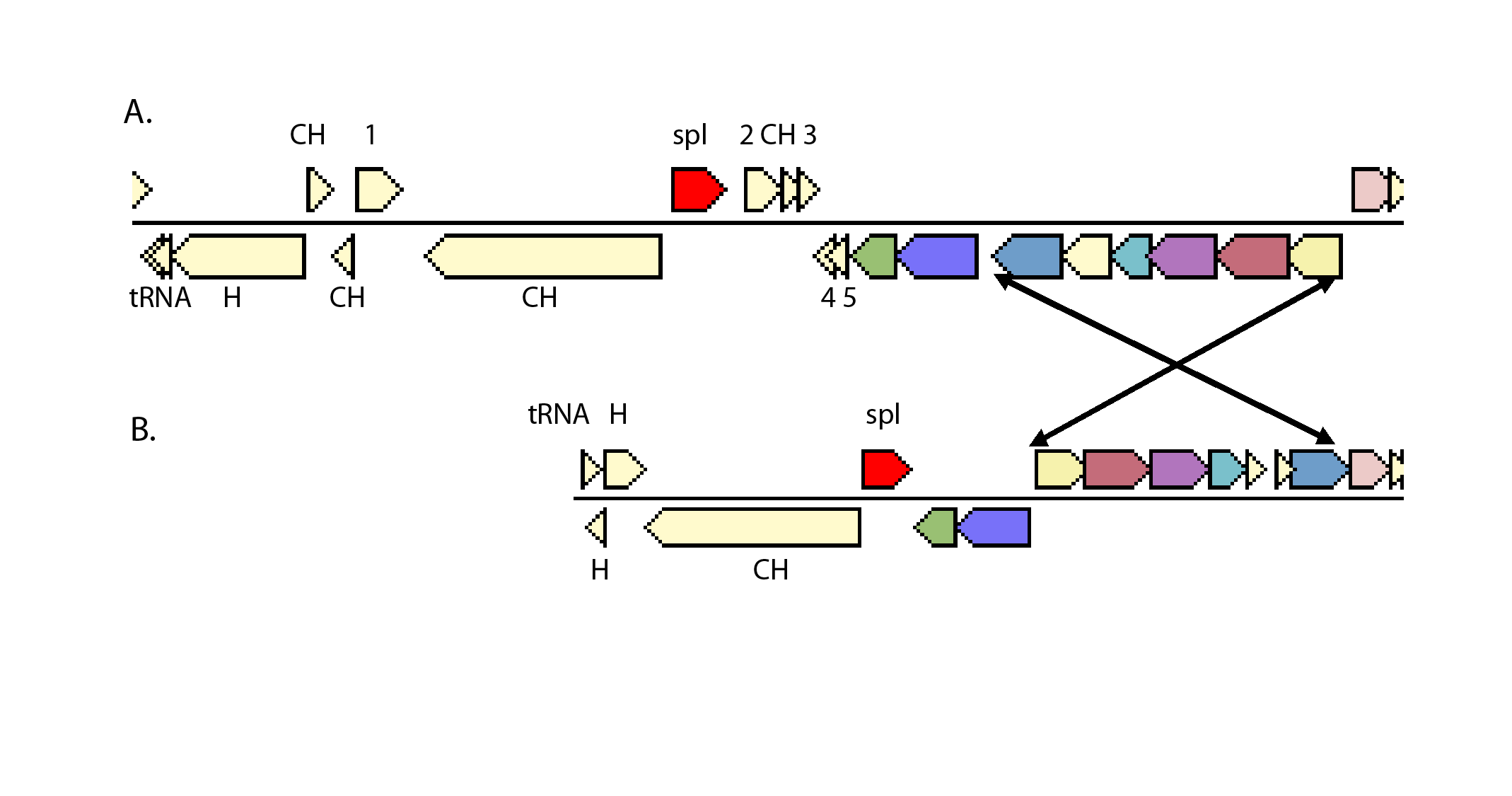

Supplement: Figure S3 — Gene neighborhoods of the spl-related gene (DNA photolyase, Radical SAM domain protein) in Nautilia profundicola (A) and Caminibacter mediatlanticus (B). Genes of the same color (except light yellow) are from the same orthologous group (top COG hit). Abbreviations: spl = DNA photolyase; H = hypothetical protein; CH = conserved hypothetical; 1 = GAMH_0510, xanthine guanine phosphoribosyl transferase; 2 = GAMH_0506, short chain dehydrogenase; 3 = GAMH_0503, plasmid stabilization system; 4/5 = GAMH_0504/502, toxin/antitoxin gene pair, RelBE-3 family. Arrows indicate a probable genome inversion between the strains. (7.78 MB TIF) [file pgen.1000362.s003.tif]
