# Supplementary material for: Adaptations to Submarine Hydrothermal Environments Exemplified by the Genome of Nautilia profundicola
Source: PLoS Genet. 2009 Feb 6;5(2):e1000362. doi: 10.1371/journal.pgen.1000362 (PMC2628731; doi:10.1371/journal.pgen.1000362)
Supplement: Table S1 — Nautilia profundicola genome sequence report as recommended by the Genome Standards Consortium [137]. (0.06 MB DOC) [file pgen.1000362.s004.doc]

Supplemental Table 1. *Nautilia profundicola* genome sequence report as recommended

| **ncbiOrganismName** :*Nautilia profundicola* strain AmH |
| --- |
| **ncbiTaxID** :244787 |
| **gcatID** :001255_GCAT |
| **goldStamp** : Gi00397 |
| **genomeProjectID** : |
| **originalSample** : |
| **collection** : |
| **organismalMaterial** : |
| **ncbiTaxID** : 6376 |
| **samplePointLocation** : East Pacific Rise |
| **LocationString** : |
| **pos2D** :12°48_7_N, 103°56_4_W |
| **marineHabitat** : |
| **waterBody** : hydrothermal vent |
| **depth** : |
| **measure** : 2500 m |
| **date** : 1999-05-30 |
| **Isolate** : |
| **publication** : |
| **articleReference** : |
| **doi** :10.1099/ijs.0.65435-0 |
| **other** :Smith et al., IJSEM, 2008 58:1598-1602 |
| **cultureCollection** : |
| **name** :ATCC |
| **identifier** :BAA-1463 (T) |
| **trophicLevel** :autotroph (producer) |
| **bioticRelationship** :free-living/opportunistic |
| **oxygenRelation** :anaerobic |
| **extrachromosomalElements** :none |
| **estimatedSize** :1,800,000 bp |
| **nucExtract** : |
| **method** :Phenol/Chloroform |
| **publication** : |
| **Reference** : Campbell et al., AEM, 2001 67:4566+ |
| **dnaLibrary** : |
| **publication** : |
| **articleReference** : |
| **doi** :DOI 10.1073/pnas.0403306101 |
| **librarySize** :2-3 kb, 8-10 kb, 10-12 kb |
| **numSequencedClones** :8170 |
| **cloneVector** : pHOS2 |
| **sequencing** : |
| **complete** : |
| **overallSequencingMethod** : Sanger dideoxy |
| **chromosome** : |
| **label** : GAMH |
| **size** : 1,676,444 |
| **gc** : 33.5% |
| **coverage** : 8x |
| **accession** : CP001279 |
| **assembly** : |
| **assemblyMethod** :Celera v.3 |
| **estimatedErrorRate** :6% |

by the Genome Standards Consortium [3].
